# Luminescent Nanocomposites of Liquid Crystals and Carbon Dots in Microfluidic Channels: Effects of Walls, Dynamics, and Bioactive Additive

**DOI:** 10.3390/nano16171122

**Published:** 2026-09-07

**Authors:** Artem Bezrukov, Aliya Galeeva, Aleksandr Krupin, Yuriy Galyametdinov

**Affiliations:** Department of Physical and Colloid Chemistry, Kazan National Research Technological University, 68 Karl Marx Str., Kazan 420015, Russia; galeeva-alija@mail.ru (A.G.); krupin_91@mail.ru (A.K.); yugal2002@mail.ru (Y.G.)

**Keywords:** nanocomposite, microfluidics, liquid crystals, carbon dots, xymedone, luminescence anisotropy, lamellar phase, hexagonal phase, artificial intelligence, neural network

## Abstract

Microfluidic confinement offers new opportunities for tailoring properties of nanomaterials by applying specific dynamic and wall effects. A vibrant approach is integration of microfluidic channels with lyotropic liquid crystals and luminescent additives, which offer a variety of tunable supramolecular organizations for applications in nanotechnology and biomedicine. This paper focuses on analyzing nanoscale and microscale properties of the nanomaterials represented by tetraethylene glycol and decaethylene glycol monododecyl ethers with integrated carbon dots. Orienting impact of microchannel surfaces was found to be responsible for additional microscale ordering of the intrinsic lamellar and hexagonal structures of these composites after the phase transition from the isotropic liquid to the liquid crystalline state. Controlled and varied shear stress resulted in additional planar orientation of the composites and provided them with anisotropic luminescence properties, which were not demonstrated by macroscopic samples. Incorporation of a bioactive compound into the liquid crystalline matrix allowed obtaining the specific and detectable anisotropic luminescence response of the nanomaterials. The datasets comprising hundreds of polarized microscopy images were successfully used for training the neural network and accurate recognition of the liquid crystal type in the composites. The results will contribute to developing AI-compatible microfluidic chips, which simulate the biological capillary environment and allow for tuning properties of luminescent nanomaterials for drug delivery and biomedical applications.

## 1. Introduction

Microfluidics is a relatively new field of science and technology, which has attracted the close and sustained attention of researchers in the last two decades [1,2,3,4]. As compared with macroscopic processes, which may cause irreproducible results in the complex chemistry of soft matter and nanomaterials [5], manipulating fluids at microscale offers sustainable and tunable conditions governed by wall effects and shear [6,7]. Therefore, microfluidic devices offer specific opportunities for steering self-organization in confinement and fabricating self-assembled materials with tailored properties [8,9].

Microfluidic devices are attractive for a variety of applications involving supramolecular chemistry and materials science: synthesis of targeted drug delivery systems [10,11,12], molecular diagnostics [13,14,15], biotechnology and biomedicine [16,17], simulating processes occurring in human organs [18,19], and synthesis and modification of functional nanoparticles and nanocomposites [20,21,22]. A novel approach is to use microfluidic devices for acquiring experimental datasets and further training of neural networks for optimizing synthesis of nanoparticles or parameters of nanoscale systems [23,24].

Liquid crystals (LC) are materials with tunable nanoscale organization that can be controlled by the orienting impact of microchannel walls and shear [6,7,25,26]. Microfluidic devices with integrated liquid crystalline materials are attractive for applications in temperature sensing [27], velocimetry [28], photonics [26], molecular detection [29,30], and biosensing [31,32]. Considering a high demand for microfluidic chips in diagnostics and medicine, biocompatible nanomaterials represented by lyotropic liquid crystals (LLC) are particularly promising [33,34,35].

Combining a liquid crystalline matrix with luminescent additives such as biocompatible nanoparticles [36,37,38] or biologically active substances [39,40] is a potent approach to developing multifunctional nanomaterials for diagnostics and medicine. Research activities of the last 10–15 years focused mostly on composites of thermotropic LC with quantum dots [41,42,43,44]. Modification of liquid crystals with biocompatible carbon dots is a more recent and actively studied approach [34,36,37,39]. As compared with thermotropic LC, lyotropic liquid crystals are mostly represented by amphiphilic molecules and offer a broader variety of nanoscale organizations depending on the structure of their polar and nonpolar groups [45,46,47,48,49]. Therefore, they can be flexible platforms for incorporated nanoparticles and pharmaceutical substances in microfluidic confinement.

The analysis of recent studies on microfluidic liquid crystal systems revealed that they are focused on thermotropic LC [6,26,29,50,51,52], while systematic studies on microfluidic lyotropic liquid crystalline systems is a newer attractive trend [33,45,53]. Currently, most publications on LC nanocomposites discuss such systems in non-microfluidic conditions [34,43,54,55,56], while research in liquid crystal nanocomposites in microchannels is represented by fewer papers, which report microfluidics as auxiliary tools and do not perform systematic studies on effect of microfluidic confinement on behavior of such systems [40,57].

This paper aims at analyzing and characterizing specific effects of microfluidic confinement on orientational and optical properties of nanocomposites formed by homologous lyotropic liquid crystals and blue carbon dots (bCD). We characterized surface properties of the nanocomposites to evaluate the effect of microchannel walls on their behavior. We analyzed the impact of the LLC type on orientational ordering and luminescent properties of the nanocomposites in static and dynamic conditions. We tested anisotropic and luminescent properties of the nanocomposites with an incorporated biologically active substance. Microfluidic datasets acquired for the studied nanocomposites allowed for training the neural network and recognizing the type of a lyotropic liquid crystal from its microfluidic texture.

## 2. Materials and Methods

### 2.1. Materials

Lyotropic liquid crystal phases were produced from tetraethylene glycol monododecyl ether—C_12_H_25_(CH_2_CH_2_O)_4_OH or C_12_EO_4_ and decaethylene glycol monododecyl ether—C_12_H_25_(CH_2_CH_2_O)_10_OH or C_12_EO_10_ (Merck, Darmstadt, Germany), 99.999% purity, which were used as received. The LLC samples were prepared by mixing 45 w.% C_12_EO_4_ or C_12_EO_10_, 5 w.% decanol, and 50 w.% water with 0.1 mg/mL of pre-dispersed carbon dots. Small additions of decanol were used according to the approach developed earlier [58] to expand the concentration range of lyotropic mesophases formed by derivatives of ethylene glycol monododecyl ethers and continue the experimental series with comparable results. The mixtures were centrifuged at 3000 rpm (25 °C) for 30 min to perform complete homogenization of the systems. Before further studies, all the samples were kept at 25 °C for 14 days.

Blue carbon dots (bCD) were synthesized by a hydrothermal method [59]. Amounts of 432 mg o-phenylenediamine and 840 mg citric acid monohydrate were dissolved in 80 mL deionized water, agitated with magnetic stirrer for 10 min, and then autoclaved in 100 mL Teflon autoclave at 180 °C for 9 h. The reaction product was cooled down and then centrifuged at 10,000 rpm for 10 min. The resulting solution was filtered with a 0.45 µm syringe filter. The filtered solution was purified with the MW 1000 dialysis membrane (where MW stands for molecular weight) in water for 24 h, and the water was changed every hour. The synthesized carbon dots were dried and used to prepare aqueous solutions.

Xymedone (N-(2-hydroxyethyl)-4,6-dimethyl-2-oxo-1,2-dihydropyrimidine, CAS number 14716-32-6) was provided by the Institute of Organic and Physical Chemistry, Kazan, Russia, with a chromatographic degree of purification. Its concentration in the aqueous phase used for preparing the composites was 1 mg/mL.

Microfluidic devices were fabricated from polydimethylsiloxane (PDMS) Sylgard^TM^ 184 silicone elastomer (Dow Corning, Midland, MI, USA). It came as a two-part elastomer kit (the pre-polymer and curing agent). SU-8 3050 photoresist (Microchem Corp., Westborough, MA, USA) was used to produce a mold for microfluidic chips.

### 2.2. Experimental Methods

Characterization of PDMS surfaces with deposited composites was performed by atomic force microscopy (AFM) using a scanning probe microscope NanoEducator by NT MDT (Moscow, Russia). Microfluidic slabs that were used for fabrication of microfluidic devices were cut to 10–10 mm rectangles after completion of the chip fabrication steps. These slabs were covered with the layer of the composites by gently shearing them with another PDMS slab and left for 3 days for drying and further AFM analysis. We analyzed heights of 8–10 LLC clusters in a series of 3 AFM images in sub-microscale and microscale ranges to calculate average height values.

Contact angles were measured by a Kruss DSA20 Easy Drop system (Kruss GmbH, Hamburg, Germany). After capturing, all the images were processed via the Kruss DSA20 Easy Drop software, version V1-03, to evaluate the contact angles. The contact angles were measured when the difference in a series of 10 consecutive measurements did not exceed ±1 degree.

The orientation behavior of the LLC media in microfluidic channels was studied by polarized optical microscopy (POM) using an Olympus BX51 microscope (Olympus, Tokyo, Japan), equipped with a high-precision Linkam heating system that allows providing uniform temperature conditions for experiments. Microscopy images were captured at 100× magnification using a ToupCam E3ISPM08300KPC camera (Touptek, Hangzhou, China).

The photoluminescence emission spectra and luminescence anisotropy data of the LLC-bCD and LLC-bCD-xymedone composites in macroscopic and microfluidic conditions were obtained by a Varian Cary Eclipse spectrofluorimeter (Agilent, Santa Clara, CA, USA) with polarization filters. The chips were placed into a holder inside the spectrofluorimeter and positioned accordingly to focus the beam on the microchannel. The spectral peaks were analyzed from at least 3 independent measurements to obtain reproducible results. The difference between the peaks did not exceed 2–3 nm.

Each luminescence anisotropy experiment included at least 3 independent measurements to obtain reproducible results. Anisotropy data were collected in the range of ±10 nm of the emission peak to obtain in total 10 points for calculating the error bars.

The luminescent properties of the LLC-bCD and LLC-bCD-xymedone composites in microchannels were studied by fluorescence microscopy using an Olympus BX43 fluorescent microscope (Olympus, Tokyo, Japan). Microscopy images were captured at 100× magnification using a ToupCam E3ISPM05000KPA camera (Touptek, Hangzhou, China).

### 2.3. Data Processing and Machine Learning Methods

Processing of the luminescence spectra and polarized microscopy images were performed by Matlab 2021b software using pre-developed customized scripts. All the respective microscopy images were taken at identical settings of both the microscope and the image capturing software (ToupView, version 4.11) supplied with the camera. The data points of the average light transmittance of the processed images demonstrate the values of the light transmittance, which were averaged by performing pixel-to-pixel analysis of microchannel’s digital images of about 500 × 500 pixels using a custom Matlab script.

The flow velocity values of the LLC media in the microchannels were evaluated by analyzing digital images of the dynamic composites using the ToupView software. The resolution of the scale bar was 10 µm.

To implement a machine learning approach for recognizing the type of the lyotropic liquid crystal from its microfluidic texture images, the Python PyTorch 2.11.0 framework was used. The characteristics of the local computer, which was used for training the neural network and processing the results, are Intel Core i7-12700 processor, 48 GB of DDR5 RAM, and a GeForce RTX 2060 graphics processor. The neural network was trained using the dataset of approximately 1000 images. Image fragments of 300 × 300 pixels were used for training.

### 2.4. Fabricating Microfluidic Devices and Performing Microfluidic Experiments

Microfluidic devices were fabricated using standard photolithography techniques [60]. Chips with rectangular microchannels of 100 µm height and the widths 100, 200, and 300 µm were fabricated. SU-8 photoresist and a transparent photomask with a negative image of a microchip were used to produce a 100 µm thick mold of microfluidic chips on top of a 3-inch silicon wafer. PDMS pre-polymer was mixed with a curing agent, poured over the mold, and allowed to cure in an oven for 4 h in 60 °C. Once cured, PDMS was peeled off the mold and bonded to a flat PDMS slab via 1 min plasma treatment using the Harrick Plasma Cleaner PDC-23G (Harrick Plasma, Ithaca, NY, USA). The PDMS device was then heated in an oven at 180 °C for 1 h to complete the bonding of the two polymer layers. The chips were fabricated with the following dimensions: 100–300 µm width, 100 µm height, and 16 mm length of the main channel.

The composite samples were infused into microfluidic devices using Shenchen ISPLab01 syringe pumps (Baoding Shenchen Precision Pump Co., Ltd., Baoding, China), which provide a minimal flow rate of 0.001 µL/min. All the microfluidic experiments were performed at 25 °C. In this work, the flow rates of the LLC materials were set in the range up to 5 µL/min. The LLC structures were observed in the middle of the main channel. The minimal residence time of the nanocomposites was about 10 s at the highest flowrate, which was sufficient to form a uniform flow pattern in the middle of the channel. To provide the same hydraulic paths for fluids to all the inlets, PTFE tubes of identical lengths (10 cm) and internal diameters that fit the same needle tips inserted into microchip outputs (20 G-type needles, 0.9 mm diameter) were used. These tubes were connected to identical 1 mL syringes installed into syringe pumps.

## 3. Results

### 3.1. Characterization of the Materials and Microfluidic Environment

**Figure 1:**
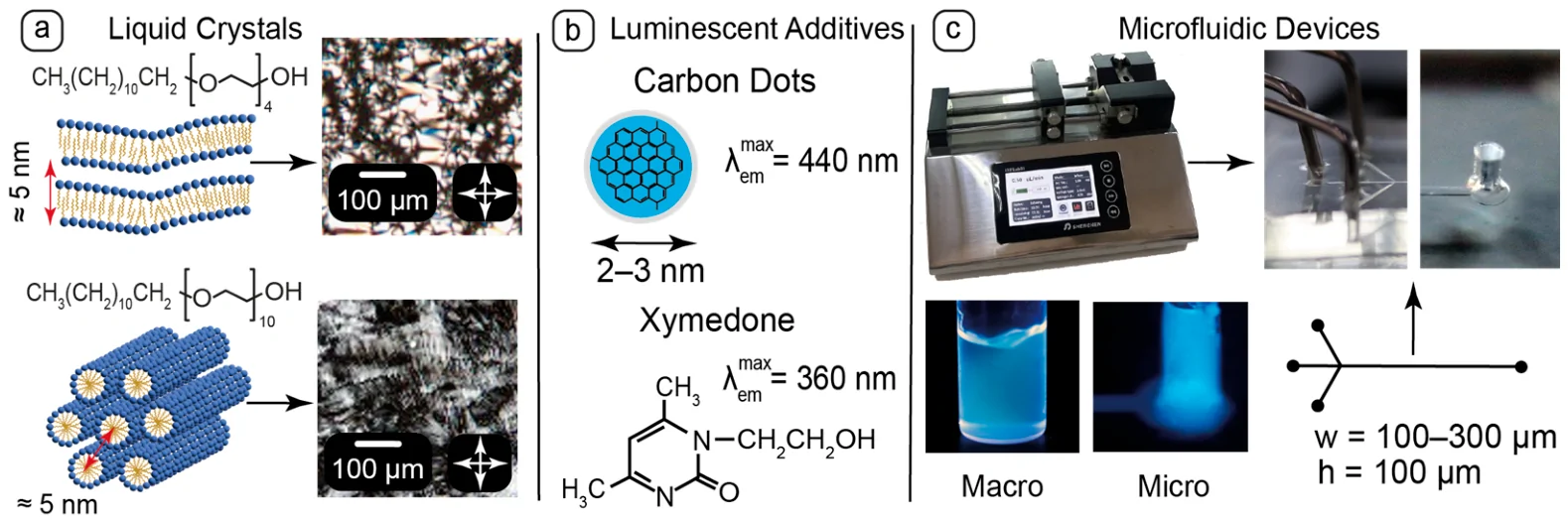
Preliminary characterization of the materials and selection of microchips: (**a**) LLC molecules, the respective mesophases, and polarized light textures in macroscopic conditions; (**b**) size and luminescence properties of carbon dots and luminescence properties of xymedone; (**c**) microfluidic devices for processing of the luminescence composites.

The first stage of this work focused on preliminary characterization of the materials and their compatibility and selecting a proper design of microfluidic devices (Figure 1).

This work continues systematic research in the LLC systems and carbon dots. In this work, we specifically provided the new data on the C_12_EO_10_ system and the LLC systems with xymedone. An important part of the work that we were eager to discuss is the comparative effect of the LLC type on the behavior of the composites, and we provided the data for the C_12_EO_4_-bCD system from the previous publications [61,62] for the reference and comparison, where it is specified.

Discussion of the current results involves the data on the structures of the LLCs and properties of carbon dots, which were reported in our earlier papers. The mesophases of C_12_EO_4_ and C_12_EO_10_ in the presence of luminescent additives were studied in [63] by X-ray diffraction (XRD). According to XRD, C_12_EO_4_ and C_12_EO_10_ form lamellar and hexagonal mesophases, respectively, represented by alternating aqueous and surfactant domains (Figure 1a) with about 5 nm interplanar distances. The structure, size, and emission properties of carbon dots (Figure 1b) were characterized in [61]. The presence of surface functional groups (–OH, –NH_2_) contributes to their good solubility in water. The fresh C_12_EO_4_ and C_12_EO_10_ LLC samples prepared for this work (Figure 1a) demonstrated the textures typical for lamellar and hexagonal mesophases. These textures agreed well with our earlier results [63] and literature [64,65].

Tetraethylene glycol and decaethylene glycol monododecyl ethers were selected for producing LLC mesophases because they represent water-soluble non-toxic surfactants forming liquid crystalline phases in water at room temperature, which is convenient for microfluidic experiments. These LLCs are characterized well in literature [65,66,67], so their behavior can be analyzed in a complex environment of microfluidic channels without the need for additional characterization.

Before microfluidic experiments, both LLC samples were studied by spectrofluorimetry and demonstrated negligible luminescent properties. Therefore, both the C_12_EO_4_ and C_12_EO_10_ LLCs can be convenient hosting matrices for luminescent dopants in luminescence studies.

Xymedone is a derivative of pyrimidine with a wide range of pharmacological activities that are related to its influence on metabolism of nucleic acids and the resulting stimulating effect on tissue regeneration and immunity [68,69,70]. This compound belongs to a broad class of biologically active substances with a pyrimidine core. It is, therefore, an attractive dopant for the biocompatible liquid crystalline matrices of C_12_EO_4_ and C_12_EO_10_ as potential drug delivery carriers. According to optical microscopy studies, both LLCs formed homogeneous composites with carbon dots and xymedone. According to spectrofluorimetry, its luminescence peak is in the near-UV range (Figure 1b). Its concentration selected for preparing the composites was sufficient to detect a luminescence response both in macroscopic and microfluidic conditions.

Microfluidic devices were fabricated from polydimethylsiloxane (PDMS) (Figure 1c), which is a biocompatible polymer with a nanoporous hydrophobic surface represented by surface CH_3_ groups [61]. Such a surface possesses anchoring capabilities for C_12_EO_4_ and C_12_EO_10_ surfactant molecules. The PDMS chips prepared for this work were tested by spectrofluorimetry and demonstrated negligible luminescence. According to spectrofluorimetry studies, a detectable and sustainable luminescence response from bCD and xymedone in the composites was provided by the chips with the main channels of 300 µm and height of 100 µm. Such chips, therefore, were selected for spectrofluorimetry experiments, while chips with smaller channels were also used for analyzing orientation behavior of the LLCs in various confined geometries.

### 3.2. Surface Characterization of the Composites in Microfluidic Channels

**Figure 2:**
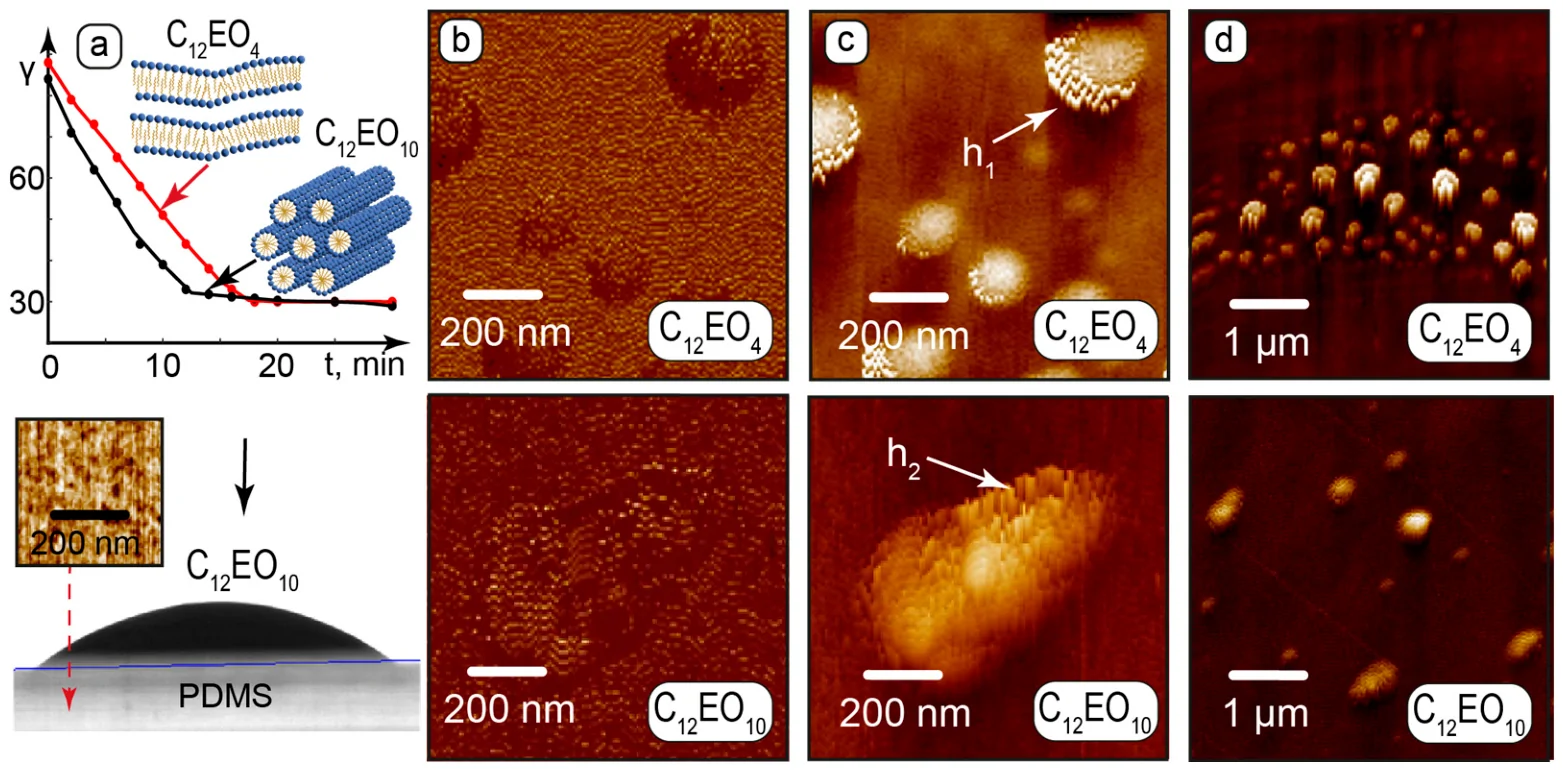
Surface characterization of PDMS with the deposited composites: (**a**) contact angles of the composites deposited on PDMS; (**b**) sub-microscale phase images of PDMS with the deposited composites; (**c**) respective sub-microscale height images of PDMS with the deposited composites; (**d**) microscale height images of PDMS with the deposited composites. LLC cluster heights: h_1_ = 87 ± 22 nm, h_2_ = 33 ± 8 nm.

As compared with macroscopic channels, a surface-to-volume ratio is much higher in microfluidic confinement. Wall effects can make, therefore, a predominant contribution to the behavior of nanomaterials in microfluidic devices. At this stage of the work, we characterized properties of the LLC-bCD nanocomposites on the PDMS surface of microchannel walls (Figure 2) by measurement of contact angles and atomic force microscopy (AFM).

The equilibrium contact angle γ ≈ 30° was reached by both C_12_EO_4_ and C_12_EO_10_ samples approximately 15–20 min after deposition of their droplets on the PDMS surface (Figure 2a). Similar wetting of the hydrophobic PDMS surface (γ ≈ 95° for water) by both LLCs can be attributed to intermolecular interactions of their identical nonpolar tails (C_12_) with CH_3_ groups of PDMS chains forming a nanoporous surface mesh (Figure 2a), while an expected orientation of polar oxyethylene fragments is perpendicular to the surface.

AFM phase imaging in the sub-microscale range (Figure 2b) revealed similar uniform surface patterns for both LLCs on PDMS and indicates that each surface layer is represented by similar molecular structures, which cover PDMS nanopores. Therefore, we can observe a definite anchoring of the LLC molecules by the PDMS surfaces, which agrees with the results of contact angle measurement.

The respective AFM height images (Figure 2c) show clusters with symmetrical nanoscale structural elements formed by both LLCs. The height of these clusters is about 87 ± 22 nm for C_12_EO_4_ and 33 ± 8 nm for C_12_EO_10_. Similar surface elements with the same average heights were observed for both LLCs in the microscale range (Figure 2d).

Higher C_12_EO_4_ clusters can be formed by lamellar blocks, which tend to additionally aggregate into sphere-like multilamellar vesicles on PDMS surface. The smaller height of the C_12_EO_10_ clusters may indicate that their hexagonal blocks are aligned by the surface and do not tend to aggregate into larger structures.

Therefore, Figure 2c,d reveals ordering in both LLCs on PDMS surfaces. We can assume a certain distortion of the patterned elements because of interactions of the cantilever with the surfaces. However, similar patterns in the nanoscale and microscale ranges demonstrate a definite alignment of LLC structural elements with respect to PDMS.

No nanoscale or microscale particles, which can represent bCD or their assemblies, were revealed by AFM studies of the composites formed by both C_12_EO_4_ and C_12_EO_10_. AFM imaging, therefore, indicates that carbon dots are uniformly distributed in these LLC matrices and do not tend to aggregate.

Thus, surface characterization of the LLC-bCD composites confirmed an important role of the wall effects in the behavior of these nanomaterials in microchannels. These effects are caused by the interactions of both C_12_EO_4_ and C_12_EO_10_ molecules with PDMS and result in their surface anchoring and symmetrical alignment of the mesophases in the nanoscale and microscale ranges.

### 3.3. Orientation Behavior of Static and Dynamic Composites in Microfluidic Confinement

Orientation ordering of liquid crystals by shear and microchannel walls influences their optical properties and can be tracked by polarized optical microscopy. In this section, we performed a comparative analysis of orientation behavior and optical properties of the C_12_EO_4_ and C_12_EO_10_ composites with bCD in static and dynamic conditions.

POM studies revealed no significant differences between the textures of the individual LLCs and their composites with bCD in all the experiments. The phase transition temperatures of the LLC and their composites were found to be similar: 43.8 °C and 45.6 °C for the C_12_EO_4_ and C_12_EO_4_-bCD composite, respectively; 38 °C and 40.2 °C for the C_12_EO_10_ and C_12_EO_10_-bCD composite, respectively. A slight increase in the clearing temperature of the composites can be attributed to the addition of bCD.

**Figure 3:**
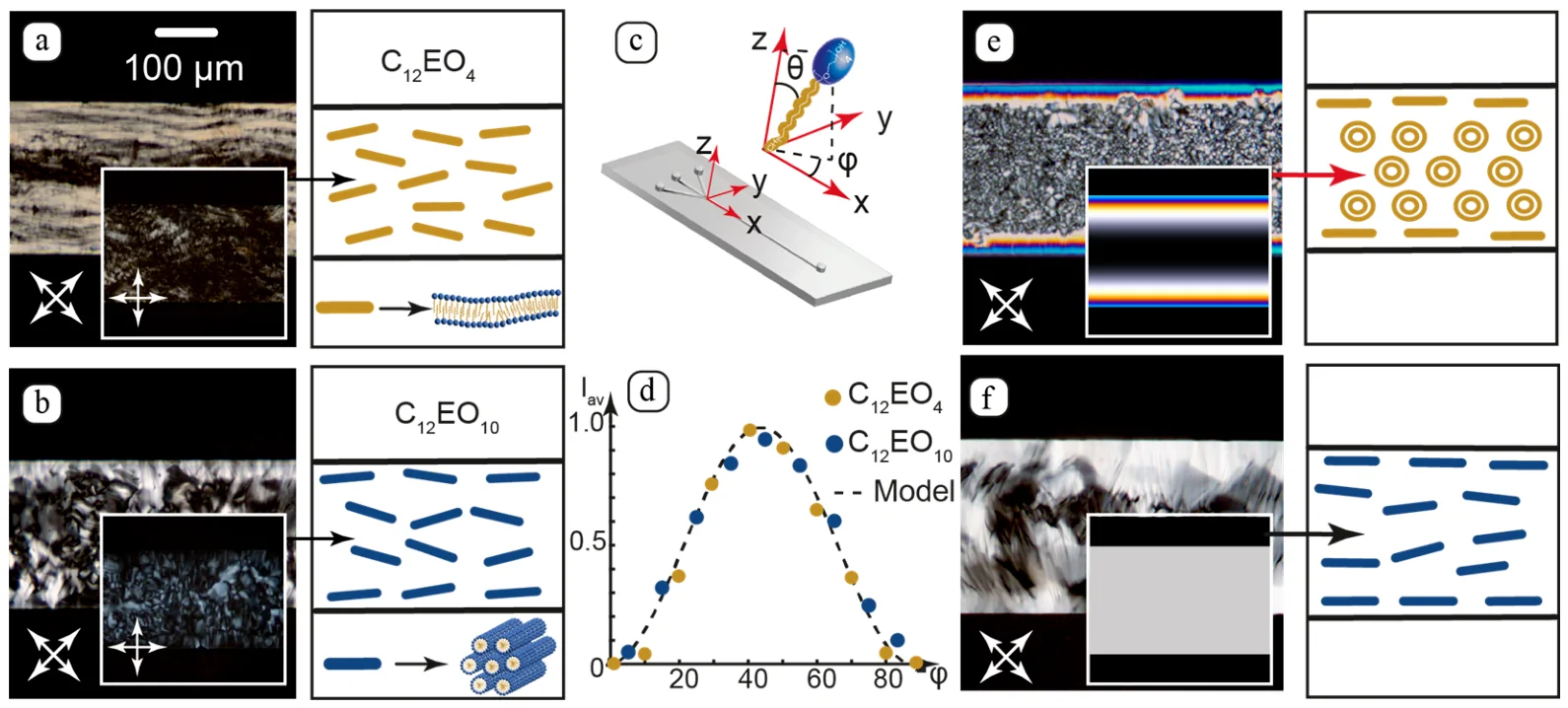
Orientation behavior of the static composites: (**a**,**b**) characteristic texture features of C_12_EO_4_ and C_12_EO_10_ mesophases, respectively, after their initial infusion into the microchannel and presumed orientation of the lamellar and hexagonal blocks; (**c**) rotation and tilt angles of LLC molecules in microchannels; (**d**) dependence of the light transmittance on the angle between microchannel walls and crossed polarizers; (**e**,**f**) textures of C_12_EO_4_ and C_12_EO_10_ mesophases, respectively, after cooling down from the isotropic liquid to 25 °C, modeling of the textures (insets), and presumed orientation of the lamellar and hexagonal blocks. Crossed arrows show positions of the polarizers. The points for C_12_EO_4_ in Figure 3d were added from the previous work [62] for comparison.

Figure 3 summarizes the orientation behavior of the static composites. The texture elements of the composites infused into the microchannels (Figure 3a,b) resemble patterns of lamellar and hexagonal mesophases formed by C_12_EO_4_ and C_12_EO_10_, respectively, in macroscopic conditions under shear [64,71].

POM data can provide information about the spatial orientation of the LLC molecules (Figure 3c) and correlate it with their self-organization in microchannels. The light transmittance and birefringence colors of microfluidic LLC textures were analyzed according to the following equation [61]:

(1) $$I=I_{0}{sin}^{2}2\varphi {sin}^{2}\frac{F}{2}$$

where I and I_0_ are the transmitted and incident light intensities, respectively, and φ is the rotation angle of LLC molecules with respect to the polarizers. $F=\frac{2\pi }{\lambda }\Delta nd$ is the phase retardation of the incident light of the wavelength λ passed through a liquid crystal layer with the thickness d and birefringence Δn.

In turn, birefringence correlates with the tilt angle θ of LLC molecules:

(2) $$\Delta n=\frac{n_{\|}n_{⊥}}{\sqrt{{n_{\|}}^{2}{cos}^{2}\theta {+n_{⊥}}^{2}{sin}^{2}\theta }}-n_{⊥}$$

where n_‖_ and n_⊥_ are anisotropic refractive indices, and θ is the angle between the optical axis of incident light and LLC molecules.

The texture images in Figure 3a,b were captured at different angles set for the crossed polarizers. Average normalized light transmittance intensities were calculated in Matlab from the POM images according to the methodology developed earlier [62] and shown in Figure 3d. They were found to fit Equation (1): $I\sim I_{0}{sin}^{2}2\varphi$ for the anchoring of LLC molecules perpendicular to microchannel walls [7]. The resulting orientation of the lamellar and hexagonal blocks, which minimizes hydraulic resistance, can be parallel to the microchannel axis.

The textures in Figure 3a,b are, however, non-uniform. We can assume, therefore, only an averaged orientation of the composites along the axis with local fluctuations. This effect can result from the initial shear alignment of the infused composites into the microchannels.

To minimize initial shear effects and obtain orientation states that are predominantly governed by microchannel walls, the composites were heated to the isotropic liquids and then cooled down to 25 °C. The resulting textures (Figure 3e,f) demonstrate a considerable effect of the microchannel walls and LLC molecular structures on their orientation.

The C_12_EO_4_ composite (Figure 3e) forms an ordered texture with lamellar onions (multilamellar vesicles) in the central part of the channel and birefringence patterns at the walls. Modeling of its texture by Equation (1) with the homeotropic orientation of the LLC molecules showed a satisfactory agreement with the experiment. Birefringence colors can be attributed to changes in the tilt angle θ of the molecules in the lamellae upon their transition from horizontal to vertical walls in the corners of the microchannel.

The C_12_EO_10_ composite (Figure 3f) shows a more ordered texture than after its initial infusion. This indicates a growing orienting effect of the walls. As compared to the C_12_EO_4_ composite, the C_12_EO_10_ composite shows no diverse birefringence colors. This effect can result from the axial symmetry of hexagonal blocks and variation of tilt angles of the constituting LLC molecules. We observe, therefore, an integral birefringence pattern of the C_12_EO_10_ hexagonal mesophase. The resulting light transmittance is supposed to be mostly governed by the rotation angle φ of its molecules in the horizontal plane. It agrees with the modeling of the texture for the planar orientation of the LLC molecules by the relation $I\sim I_{0}{sin}^{2}2\varphi$ without considering their tilts θ.

A similar impact of the microchannel walls on the composites was observed in microchannels of different widths (100–300 µm) and geometries (straight and serpentine). The results agree with contact angle and AFM data and confirm orientation of the LLC molecules in both composites perpendicular to microchannel walls.

**Figure 4:**
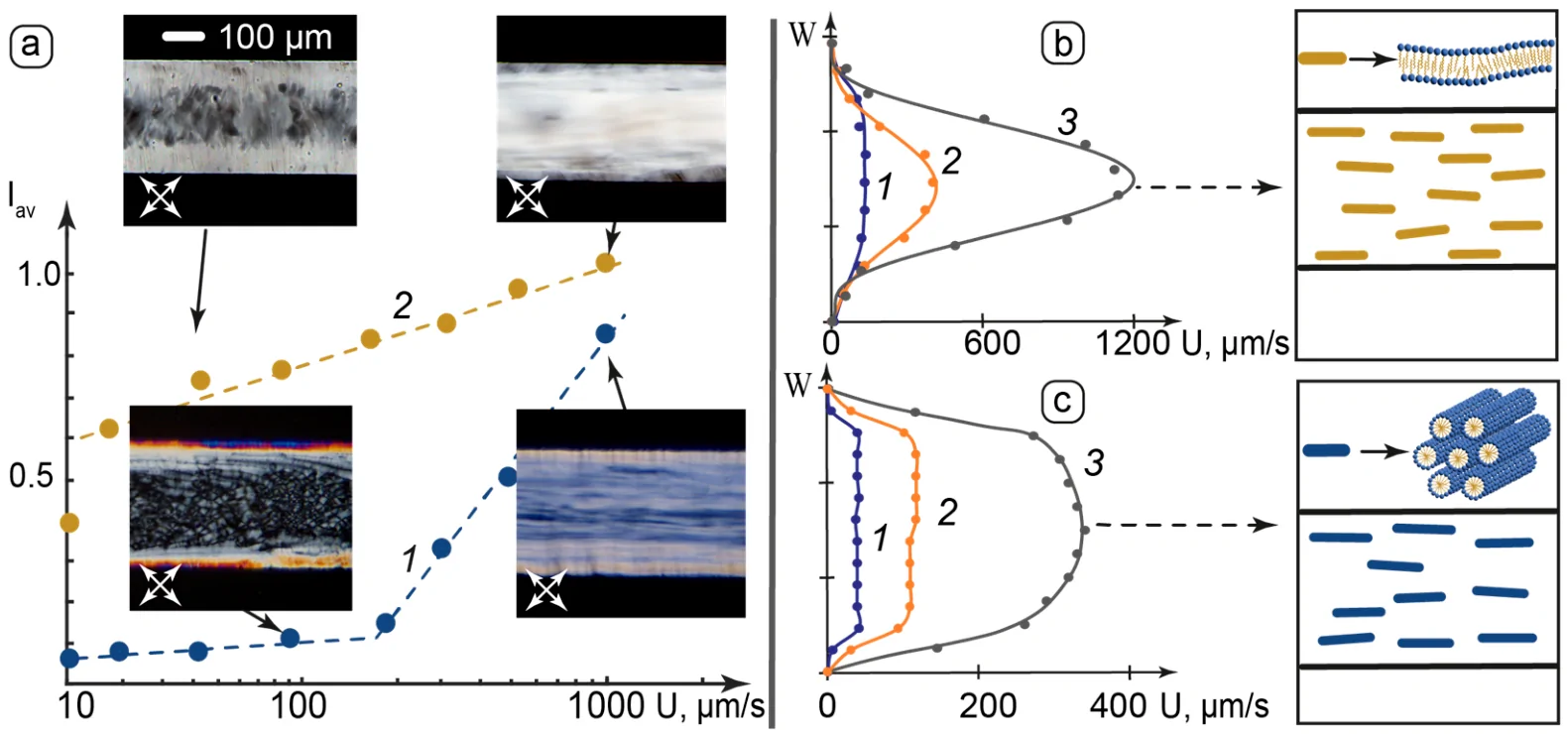
Orientation behavior of the dynamic composites: (**a**) the light transmittance at crossed polarizers and the textures of the dynamic C_12_EO_4_ (1) and C_12_EO_10_ (2) composites; (**b**,**c**) flow velocity profiles of the C_12_EO_4_ and C_12_EO_10_ composites at the flowrates set by the pump, 1 µL/min (1), 2 µL/min (2), and 4 µL/min (3) and presumed orientation of the lamellar and hexagonal blocks at the highest flow velocity. Crossed arrows show positions of polarizers.

In addition to wall anchoring, flow dynamics is another key microfluidic factor, which allows precise control of the orientation behavior of liquid crystals by performing their shear-induced structural transformations [7]. The flows of the composites in microchannels were studied by POM. The results are shown in Figure 4.

Figure 4a represents the dependence of the average normalized light transmittance of the flow velocity. The flow velocity values were evaluated by analyzing digital images of the dynamic composites using the ToupView software. The resolution was 10 µm.

The light transmittance of the dynamic C_12_EO_4_ composite represents two flow stages, which correspond to decomposition of lamellar onions into axial lamellar structures and further flow ordering of the lamellar threads. The light transmittance of the dynamic C_12_EO_10_ composite in microchannels demonstrates considerable growth after the initial weak shear. This effect is attributed to the initial shear ordering of the hexagonal blocks along the channel axis and agrees with literature [64].

At high flow velocities, the composites show similar textures, which can represent predominant axial orientation of their lamellar and hexagonal blocks.

The analysis of the shifts of the dynamic LLC texture elements at different points of microchannels allowed plotting the flow velocity profiles of the composites. Figure 4b,c shows these profiles for specific flow rates set by the syringe pump, which are proportional to the pressure applied to microchannel inlets. At the low set flow rate, the flow velocities of both the composites are uniform across the microchannels, indicating high shear resistance of the static structures formed by interacting lamellar and hexagonal units.

At higher set flow rates, we can observe a gradual transformation to flow velocity distributions that are similar to Poiseuille flow. In the C_12_EO_4_ composite, this effect can be related to the decomposition of static structural elements represented by lamellar onions. In the C_12_EO_10_ composite, this effect can be related to the axial re-alignment of the microscale domains formed by hexagonal blocks and further decomposition of these domains into smaller dynamic units.

The flow-induced transformations of the composites were found to be reversible. Applying a heating-cooling cycle with the phase transition to isotropic liquid and further cooling down to room temperature allows returning to the non-sheared textures shown in Figure 3e,f and repeat flow-induced transformation of the composites.

Similar flow behavior of the composites was observed in microchannels of different widths (100–300 µm) and geometries (straight and serpentine).

Thus, wall effects and flow dynamics in microchannels allowed performing controlled transitions between supramolecular organizations of the studied composites. The LLC systems demonstrated a structural evolution under different flow conditions. At zero flow, the LLC molecules in both composites are aligned perpendicular to microchannel walls that induce additional orientation of the lamellar and hexagonal blocks parallel to the horizontal and vertical walls of the microchannel. Applied shear induces reorientation of these blocks along the flow axis and decomposition of the microscale LLC domains, which is accompanied by development of parabolic flow profiles in both LLC composites.

### 3.4. Luminescent Properties of the Liquid Crystal Composites with Carbon Dots and Xymedone

**Figure 5:**
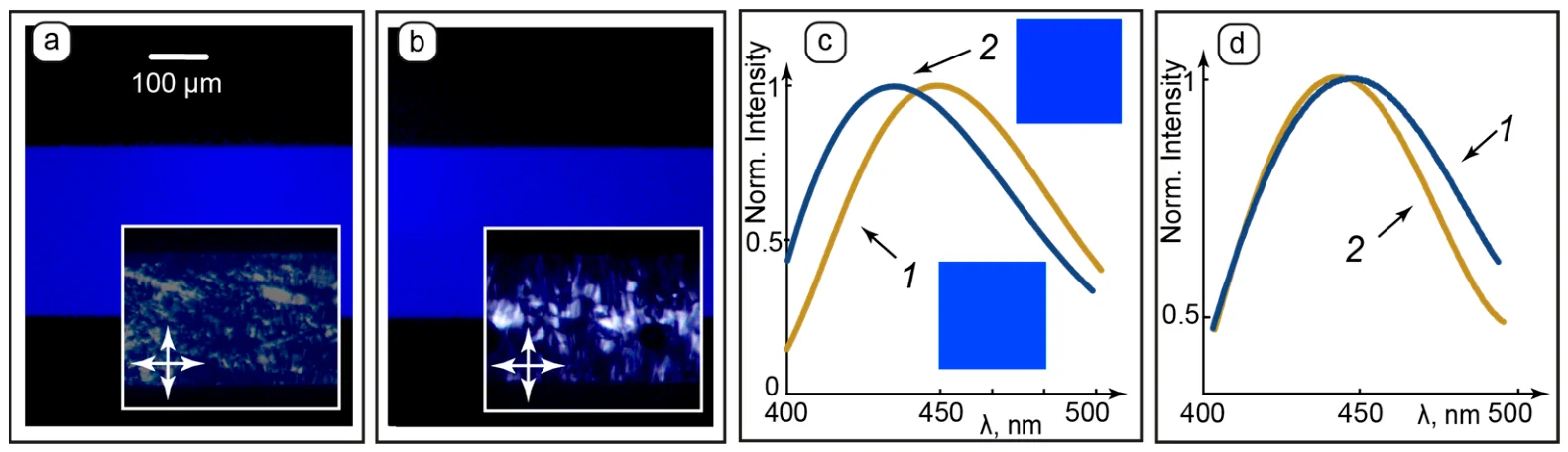
Luminescent properties of the composites: (**a**,**b**) fluorescence microscopy images of the C_12_EO_4_ and C_12_EO_10_ composites, respectively, in microchannels and their textures at crossed polarizers (insets) with arrows representing positions of the polarizers; (**c**) normalized emission spectra of the C_12_EO_4_ (1) and C_12_EO_10_ (2) composites in cuvettes, square insets represent the emission colors corresponding to the spectra; (**d**) normalized emission spectra of the C_12_EO_4_ (1) and C_12_EO_10_ (2) composites in microchannels.

Hybrid anisotropic and luminescent organized media are promising materials for medical diagnostics and targeted drug delivery applications [34]. In this section, we discussed luminescent properties of the C_12_EO_4_ and C_12_EO_10_ composites with bCD and xymedone in macroscopic and microfluidic conditions. Fluorescence microscopy and spectroscopy data of the nanocomposites with bCD are shown in Figure 5.

Both the composites generated stable, intensive, and homogeneous blue color emission in microchannels of all the studied geometries at all the studied set flowrates (Figure 5a,b). These results agree with AFM, which demonstrated no microscale aggregation of bCD in both the composites. The emission was also found to be uniform across the microchannels. Therefore, no considerable wall effects on luminescence of bCD were detected, and their distribution in the LLC matrices of the composites can be more preferable than interactions with hydrophobic PDMS surfaces due to the hydrophilic nature of the studied carbon dots and their possible location in aqueous domains of the mesophases.

The emission spectra of the macroscopic composites show a slight shift of the peak for C_12_EO_10_ to a shorter wave area. This effect can result from possible interactions of bCD polar surface groups with oxyethylene groups in C_12_EO_10_ molecules, which are larger than those of C_12_EO_4_ molecules, via hydrogen bonding [72,73]. Modeling the emission color from the spectra according to the methodology reported earlier [74] predicts a similar blue color emission from both the composites, which agrees with fluorescence microscopy images. No considerable changes in the emission spectra of the composites were observed after their infusion into microchannels (Figure 5d). However, it was challenging to detect a sustainable peak shifting effect in microfluidic conditions that can be attributed to a more complex microchannel environment, possible scattering effects, and optimization of excitation conditions for each sample.

An attractive feature of luminescent media in biomedical applications and molecular analysis is their capability for anisotropic light emission [75]. The luminescence anisotropy of bCD in the composites was evaluated from the polarized spectrofluorimetry data as described in [61]. The results are summarized in Table 1.

Both macroscopic and microfluidic composites demonstrate similar emission peaks as compared with aqueous bCD. Therefore, infusion of the composites into microchannels is not supposed to substantially change the surrounding environment of bCD. Similar luminescence behavior of aqueous bCD and bCD in the composites based on C_12_EO_4_ and C_12_EO_10_ also indicates their similar surrounding environment, which may be represented by the aqueous domains in lamellar and hexagonal matrices, respectively.

The composites showed negligible anisotropic luminescence properties in macroscopic conditions. Incorporation of the composites into microchannels provided them with luminescence anisotropy in static and dynamic conditions, which grows with applied shear for both the composites. The luminescence anisotropy of the samples sheared at the flow rates of 2 and 4 µL/min did not demonstrate considerable changes in the luminescence anisotropy. This effect may indicate completed alignment of the structural elements of the mesophases at the studied flow rate of 2 µL/min. The bCD + C_12_EO_10_ composite demonstrates higher capabilities for anisotropic luminescence, which can be attributed to its higher ordering by microchannel walls and shear. The results agree with AFM and POM data and indicate that ordering of their LLC matrices in microchannels can contribute to anisotropic luminescence properties of the studied LLC-bCD composites.

To summarize, carbon dots are uniformly distributed in both the LLC matrices and do not tend to aggregate in static and dynamic conditions according to fluorescence microscopy studies and AFM data. According to fluorescence spectroscopy data, the emission spectra of bCD do not undergo considerable changes after incorporation of these nanoparticles into the LLC matrices. Hydrophilic carbon dots are, therefore, expected to be located in aqueous domains of the LLCs and do not undergo strong interactions with LLC molecules, which can result in changes in the fluorescence spectra or provide a notable anisotropy of luminescence in macroscopic non-aligned composites. Non-zero anisotropy of aqueous bCD in microchannels can be attributed to optical peculiarities of microfluidic devices. The microfluidic spectrofluorimetry technique is planned to be further elaborated and optimized in future studies. In microchannels, both studied LLCs form anisotropic matrices aligned with respect to microchannel walls and axis. Growth in the luminescence anisotropy of the composites after infusion in microchannels and applied shear correlates with the structural evolutions of both the LLC matrices. Therefore, the structural alignment of the studied anisotropic liquid crystals in microfluidic confinement makes a notable contribution to the anisotropic optical response of bCD.

**Figure 6:**
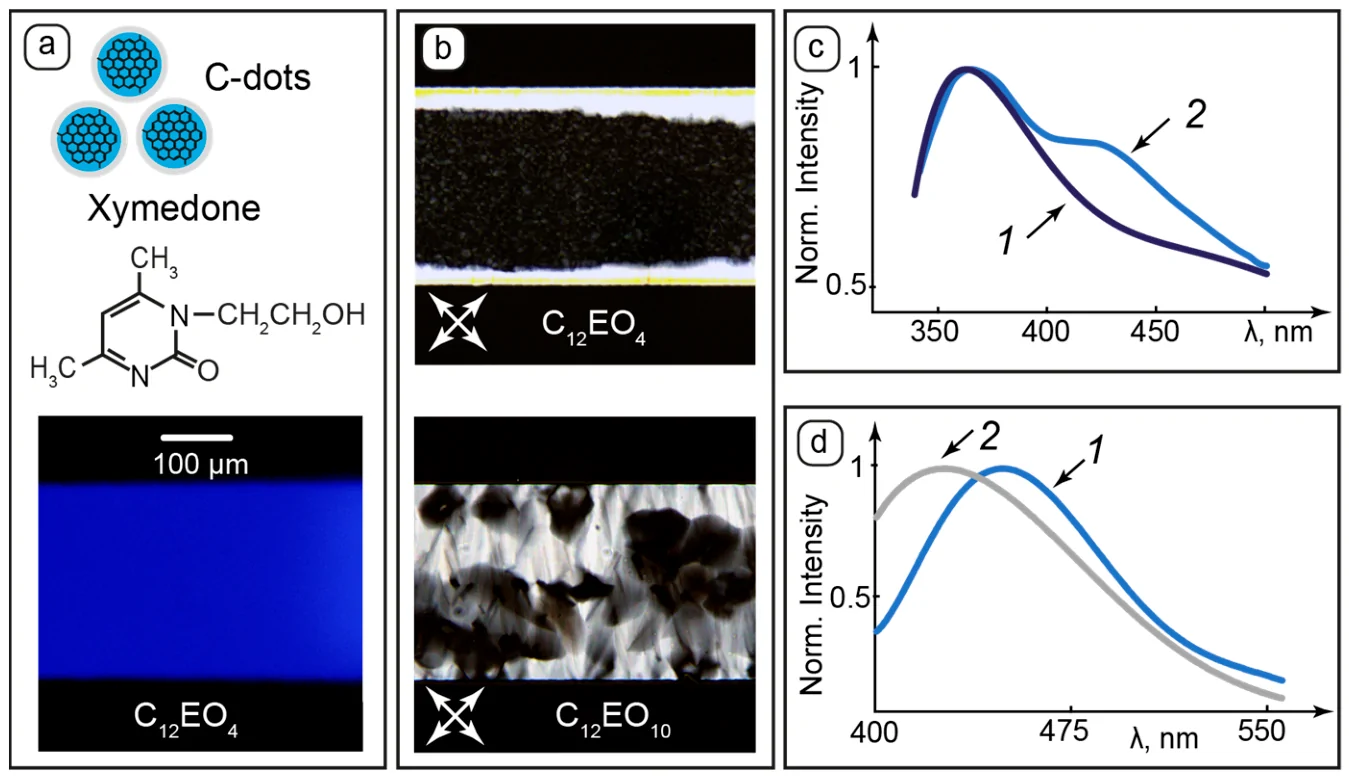
Orientation and luminescent behavior of the composites with xymedone: (**a**) an example of similar fluorescence microscopy images represented by the Xym + bCD + C_12_EO_4_ composite; (**b**) POM images of the Xym + bCD + C_12_EO_4_ and Xym + bCD + C_12_EO_10_ composites; (**c**) normalized emission spectra of the aqueous xymedone (1) and the Xym bCD + C_12_EO_4_ composite (2) for λ_ex_ = 325 nm; (**d**) normalized emission spectra of the bCD + C_12_EO_4_ (1) and Xym + bCD + C_12_EO_4_ (2) composites for λ_ex_ = 370 nm. Crossed arrows show positions of polarizers.

Biomedical applications of nanomaterials assume their compatibility with biologically active compounds. In this respect, we performed initial studies of the orientation behavior and luminescence properties of the Xym-LLC-bCD composites, which are summarized in Figure 6. Additions of this biologically active component did not result in considerable changes in the fluorescent microscopy images (Figure 6a) and in the LLC textures (Figure 6b). Therefore, xymedone was found to be compatible with the LLC matrices and their composites with carbon dots, and its addition is not supposed to deteriorate their intrinsic lamellar and hexagonal ordering and luminescent properties in macroscopic and microfluidic conditions.

The effect of added xymedone on the clearing temperature was found to be more considerable than that of bCD. It increased the clearing temperatures of the C_12_EO_4_ and C_12_EO_10_ composites to 51.1 °C and 46.8 °C, respectively. This effect can be attributed to possible interactions of xymedone molecules with the LLC matrices.

The components of the Xym-LLC-bCD composites were found to exert a mutual influence on their luminescent properties according to macroscopic studies. The emission spectrum of the aqueous xymedone (Figure 6c) is in the near-UV range. The emission spectrum of the Xym-C_12_EO_4_-bCD composite shows an additional shoulder in the longer wave area, which corresponds to the maximum emission intensity range of bCD (Figure 5c). This effect can be explained by the fact that the emission maximum of xymedone is close to the excitation maximum of carbon dots, so luminescence of the drug can also initiate emission from bCD.

On the other hand, the emission spectrum of carbon dots in the composite shifts slightly to a shorter wave area (Figure 6d) in the presence of xymedone, which can be related to possible interactions between xymedone and bCD in the LLC matrix.

Introduction of both the composites with xymedone into microchannels found that they maintained their luminescence behavior and provided them with additional anisotropic luminescence properties with respect to xymedone (Table 2). Similar effects were observed for Xym-C_12_EO_10_-bCD composites in macroscopic and microfluidic conditions.

Xymedone demonstrates similar excitation and emission peaks and the respective spectra in macroscopic and microfluidic conditions that can be explained by a similar surrounding environment in lamellar and hexagonal mesophases. As opposed to bCD, xymedone exhibits non-zero luminescence anisotropy in the macroscopic composites, which may also indicate its possible interactions with the hosting environment represented by the LLC matrices and bCD and agrees with the spectral data in Figure 6c,d.

Evaluation of luminescence anisotropy in microfluidic confinement resulted in larger baseline luminescence anisotropy values in all the experiments with bCD and xymedone as compared to “macroscopic” experiments in a cuvette. This effect was sustainable and reproducible. We can attribute this effect to a possible impact of optical properties of microfluidic devices on the resulting data calculated by the spectrofluorimeter’s software, which is by default optimized to cuvettes. Further optimization of luminescence anisotropy measurements to microfluidic devices will be a part of our future research.

The major reason behind our decision to add luminescence anisotropy data to this work was the fact that we detected a reproducible growth in the luminescence anisotropy values in microfluidic channels for bCD-LLC samples as compared to microfluidic aqueous solutions systems of these luminescent additives. Considering the fact that the luminescence anisotropy of these composites is near zero in a cuvette, we can conclude that the LLC matrix in microchannels makes a certain contribution to the luminescence anisotropy of the composites, which can be related to its alignment by microchannel walls and shear.

Dynamic composites also show a growth in the luminescence anisotropy for xymedone. Therefore, anisotropic luminescence properties of xymedone in the microfluidic composites are provided by the contributions from its positioning in the LLC matrices and orientation of the mesophases in the microchannels.

Thus, the LLC composites with bCD and xymedone were proved to be compatible with microfluidic devices and maintained their orientation behavior and luminescence properties in microchips. Dynamic and wall effects of the microfluidic confinement provided the studied nanomaterials with additional anisotropic luminescence capabilities.

### 3.5. Application of Machine Learning Tools to Recognizing the Type of Liquid Crystal from Its Microfluidic Textures

**Figure 7:**
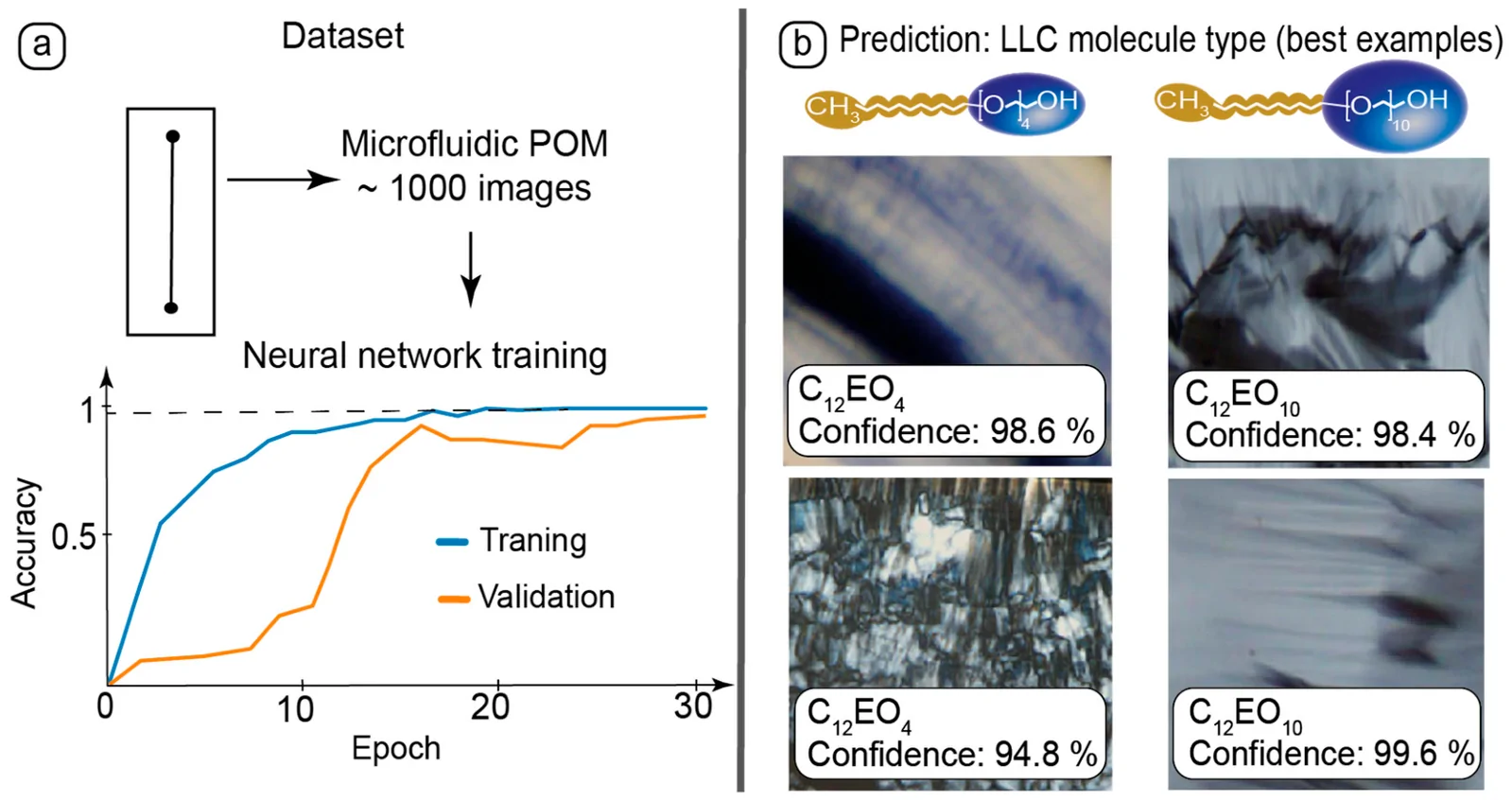
Application of AI tools to recognizing the type of liquid crystal: (**a**) training neural network with the dataset of approximately 1000 POM images of C_12_EO_4_ and C_12_EO_10_ mesophases; (**b**) using training results for predicting LLC molecule type from the texture images.

Artificial intelligence instruments can be applied for recognition of the molecular structure of materials from their specific morphology in microscopy images [76]. Microfluidic samples of the composites studied by polarized optical microscopy were found to form a variety of specific textures, which depended on the molecular structures of the LLCs and were not observed in macroscopic conditions. In the final part of this work, the POM data obtained for C_12_EO_4_ and C_12_EO_10_ mesophases were combined into datasets and used for neural network training. The training results and LLC type recognition tests are summarized in Figure 7.

Experimental datasets were collected by capturing about 1000 POM images of the composites in static and dynamic conditions in various parts of straight and curved microchannels (Figure 7a) to obtain the data points representing different images. The dataset provided sufficient training accuracy and validation.

After training, the neural network was tested with new images representing textures formed by different LLC molecules (Figure 7b). The neural network correlated the LLC molecular structure with the textures of the respective samples in polarized light.

The test set was 20% of the POM images. The training accuracy reached the plateau at 0.95. The overall classification accuracy was 70–80%, which demonstrates the good potential of this approach for processing large datasets of microfluidic images.

Therefore, the machine learning approach was successfully applied to recognition of the LLC molecular structure by analyzing their textures in polarized light in microchannels of different geometries.

## 4. Discussion

In this work, we revealed and characterized the role of microfluidic confinement in regulating liquid crystal alignment and its influence on luminescence anisotropy. Interactions of amphiphilic liquid crystal molecules with microchannel surfaces resulted in their perpendicular wall anchoring, which was enhanced by eliminating initial shear effects by heating to isotropic liquid and further cooling down. Applied shear induces a gradual and controlled realignment of the structural LLC units along the channel axis. These shear and wall effects imposed additional ordering of both lamellar and hexagonal mesophases in the LLC composites with carbon dots and xymedone. A combination of wall anchoring and shear was found to additionally contribute to luminescence anisotropy of the studied LLC nanomaterials.

We demonstrated that similar lyotropic liquid crystalline materials are capable of creating a variety of specific orientation states in microfluidic confinement, which are governed by differences in their molecular structure. Increase in the size of the oxyethylene group resulted in a change of the intrinsic molecular packing from lamellar to hexagonal. This initial packing remained stable after addition of the studied amounts of carbon dots and xymedone both in macroscopic conditions and microfluidic confinement.

Orientation states demonstrated by liquid crystals with different molecular structures in microfluidic channels were found to be more diverse as compared to non-microfluidic conditions. This allowed for training a neural network to recognize the type of LLC molecule from its microfluidic POM images.

We performed a neural network processing as a demonstrative test, which shows that it can be used to distinguish LC types from its microscopy images. The results of this work show the potential of conjugating microfluidics with neural network processing. Firstly, liquid crystals form a much broader variety of different textures in microchannels as compared to “macroscopic” conditions. Microchips, therefore, can be used to generate uniform datasets in a small number of experiments and train neural networks to recognize types of liquid crystals and molecular alignment. On the other hand, this approach will be developed in future research for classification of more types of thermotropic and lyotropic liquid crystals and their composites with quantum and carbon dots in datasets with a higher overall classification accuracy and a focus on performing real-time recognition of microfluidic samples.

The revealed effects highlight the following possible applications of the studied nanomaterials in confinement: development of lab-on-chip devices with controlled polarized luminescence from biocompatible luminescent markers, testing drug delivery systems with various hosting lyotropic mesophases, analyzing behavior of amphiphilic materials in models of biological capillary systems, and application of AI instruments to processing experimental data. Optical modulation using composite materials and microfluidic confinement broaden the application context of such nanosystems to such areas as inkjet printing with nanoparticles [77], nanospheres for sensing and interactive displays [78], and anti-counterfeiting with luminescent nanoparticles [79].

We studied LLC composites with xymedone in macroscopic and microfluidic conditions for possible biomedical applications of such materials. LLC-bCD-Xym nanocomposites can be luminescent theranostic platforms with controlled release characterization capabilities in microfluidic conditions. Successful incorporation of this drug with the LLC matrix and carbon dots in microchannels highlights deeper research plans in the future for LLC composites with xymedone. These plans include the dose-dependent studies of LLC composites with xymedone and characterizing the optical parameters of the composites in macroscopic and microfluidic conditions in future studies and characterizing the behavior of xymedone in mesophases to evaluate the application potential of the studied composites as drug-carrying matrices with the use of AI tools for processing microscopy images and spectrofluorimetry data.

## 5. Conclusions

The studied microfluidic mesophases demonstrated specific orientation states, which depended on the size of the oxyethylene group and were governed by microfluidic confinement. A combination of wall anchoring, temperature, and shear effects allowed achieving controlled and reversible ordering of the lamellar and hexagonal blocks perpendicular to microchannel walls and parallel to the microchannel axis and performing a quantitative control and computer modeling of the light transmittance and birefringence of the microfluidic mesophases. Incorporation of carbon dots and xymedone into the mesophases was found to maintain their intrinsic lamellar and hexagonal packing, additional ordering, and luminescence properties both in macroscopic conditions and in static and dynamic confinement. Microfluidic channels provided these composites with additional controlled anisotropic luminescence properties governed by wall anchoring and shear. The obtained microfluidic datasets allowed training the neural network to recognize the type of liquid crystal molecules from their microfluidic images.

The orientation and luminescence effects of the studied composites highlight their applications as anisotropic luminescent nanomaterials for microfluidic photonics and luminescent systems for testing incorporation and release of biologically active substances in AI-powered microfluidic devices representing models of biological capillary systems.

## Figures and Tables

**Table 1:** Luminescent properties of carbon dots and the nanocomposites.

Conditions	System	Q_set_, µL/min	Emission Peak **, nm	Anisotropy
Cuvette	bCD + H_2_O *	-	449	0.02 ± 0.01
Cuvette	bCD + C_12_EO_4_ *	-	450	0.02 ± 0.01
Cuvette	bCD + C_12_EO_10_	-	433	0.02 ± 0.01
Chip	bCD + H_2_O *	-	442	0.08 ± 0.03
Chip	bCD + C_12_EO_4_ *	0	440	0.16 ± 0.03
Chip	bCD + C_12_EO_4_ *	2	443	0.25 ± 0.04
Chip	bCD + C_12_EO_4_	4	442	0.26 ± 0.02
Chip	bCD + C_12_EO_10_	0	450	0.20 ± 0.03
Chip	bCD + C_12_EO_10_	2	451	0.30 ± 0.04
Chip	bCD + C_12_EO_10_	4	450	0.32 ± 0.03

* The values for aqueous bCD and bCD + C_12_EO_4_ system were added from the previous work [61] for reference. ** The excitation wavelength was 375 nm in all the experiments.

**Table 2:** Luminescent properties of xymedone and the nanocomposites.

Conditions	System	Q_set_, µL/min	Emission Peak, nm	Anisotropy
Cuvette	Xym + H_2_O	-	360	0.07 ± 0.03
Cuvette	Xym + bCD + C_12_EO_4_	-	361	0.15 ± 0.02
Cuvette	Xym + bCD + C_12_EO_10_	-	360	0.2 ± 0.03
Chip	Xym + H_2_O	0	350	0.15 ± 0.03
Chip	Xym + bCD + C_12_EO_4_	2	352	0.28 ± 0.03
Chip	Xym + bCD + C_12_EO_10_	2	350	0.35 ± 0.05

## Data Availability

The original contributions presented in this study are included in the article. Further inquiries can be directed to the corresponding author.

## Abbreviations

The following abbreviations are used in this manuscript:

LC	liquid crystal
LLC	lyotropic liquid crystal
bCD	blue carbon dots
Xym	xymedone
POM	polarized optical microscopy
C_12_EO_4_	tetraethylene glycol monododecyl ether
C_12_EO_10_	decaethylene glycol monododecyl ether
PDMS	polydimethylsiloxane
AI	artificial intelligence
